# The first report of tinea nigra from Iran

**DOI:** 10.1016/j.nmni.2022.101032

**Published:** 2022-09-20

**Authors:** A. Kelarestaghi, S.J. Hashemi, Z. Rafat, H. Kelarestaghi, Z. Rastgar Moqaddam, A. Maboudi, S. Khodavaisy

**Affiliations:** 1) Medical Mycology Specialized Unit, Central Laboratory, Mazandaran University of Medical Sciences, Noor, Iran; 2) Department of Medical Parasitology and Mycology, School of Public Health, Tehran University of Medical Sciences, Tehran, Iran; 3) Department of Medical Microbiology, School of Medicine, Guilan University of Medical Sciences, Rasht, Iran; 4) Student Research Committee, Iran University of Medical Sciences, Tehran, Iran; 5) Private Practitioner, Amol, Mazandaran, Iran

**Keywords:** *Hortaea werneckii*, Iran, Superficial mycosis, Tinea nigra

## Abstract

*Hortaea werneckii* causes Tinea nigra, a rare superficial mycosis. It has not been reported in Iran yet. We report a case of an Iranian boy resident of Amol (Mazandaran, Iran) that developed brown macules on his left palm. Direct microscopic examination and culture confirmed the diagnosis of Tinea nigra.

## Introduction

Tinea nigra is an infrequent superficial mycosis caused by the geophilic halotolerant dematiaceous fungus *Hortaea werneckii* (formerly known as *Cladosporium werneckii*, *Phaeoannellomyces werneckii*, and *Exophiala werneckii*, and) [1]. It usually manifests as unilateral or asymmetrical brown or black patches on the palmar surface of the hand or finger (more commonly) or on the plantar surface of the foot, although it can also affect the neck or trunk [2]. The patches have irregular shapes with a darker border and tend to get bigger over time and they mimic silver nitrate stains in appearance. As a result of infection of the stratum corneum layers of the epidermis, associated lesions are usually flat and black. They are slightly scaly and do not itch or sting. The majority of patients have no obvious specific predisposing factors, and immune system deficiency does not appear to be relevant. There is no link between the genetic as a predisposing factor. The majority of infected individuals have hyperhidrosis or have lived or traveled in tropical locations [2,3]. Animals have not been affected by this infection. It is endemic in tropical and subtropical regions and often affects young people, mainly children, who get in contact with soil. This chronic infection can be misdiagnosed as malignant melanoma or a junctional melanocytic nevus and unnecessary biopsies may be performed [4]. Thus, dermoscopy can be used to distinguish tinea nigra from melanocytic lesions. The first authentic description of the disease was made by Manson in 1872 in Southern China [5]. However, so far there is no report of tinea nigra from Iran. The aim of this article was to describe the first report of tinea nigra in Iran, as well as the epidemiological, clinical, and therapeutic aspects of the disease.

## Case report

**Fig. 1:**
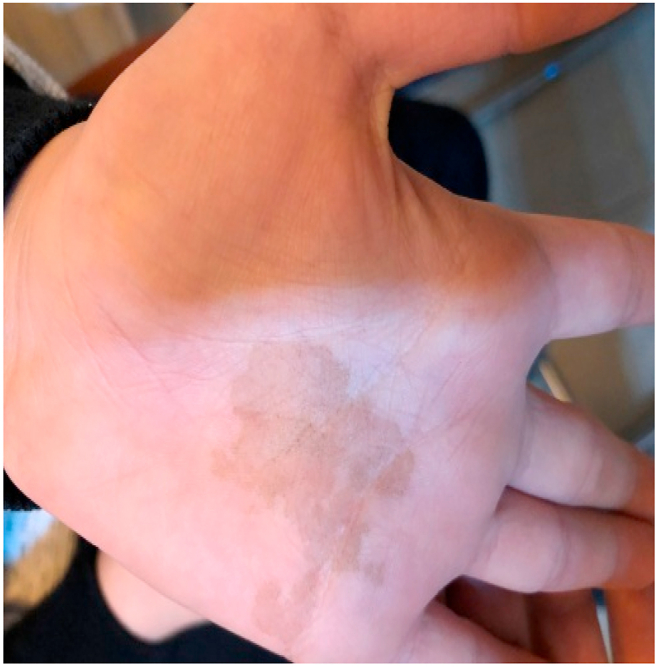
Pigmented macule on the left palm.

A 9-year-old rural boy from Amol, Mazandaran, northern Iran presented with a 2-year history of a lowly growing asymptomatic hyperpigmented macules on his left palm. He denied pruritus or pain and no other parts of the body were affected. He had no relevant history of trauma or underlying disorders. On physical examination, a brown four-centimeter macula, with irregular pigmentation and shape; it had defined edges, without scales or symptoms of inflammation (Fig. 1). There was no history of a similar condition in the family.

**Fig. 2:**
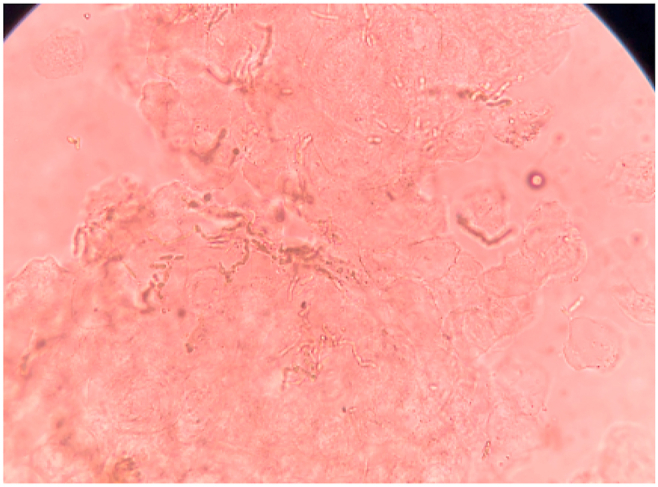
KOH preparation in microscopic observation.

The patient's lesional skin scrapings were taken. For direct microscopic examination, the specimen was prepared in KOH 10% solution and observed under a microscope. The skin scrapings revealed numerous branching and septate hyphae of various diameters when examined under the microscope. The hyphae's apical part was hyaline, with parallel walls and regular septation. The oldest sections have a brownish color, a wavy cell wall, and irregularly distributed septa. (Fig. 2).

**Fig. 3:**
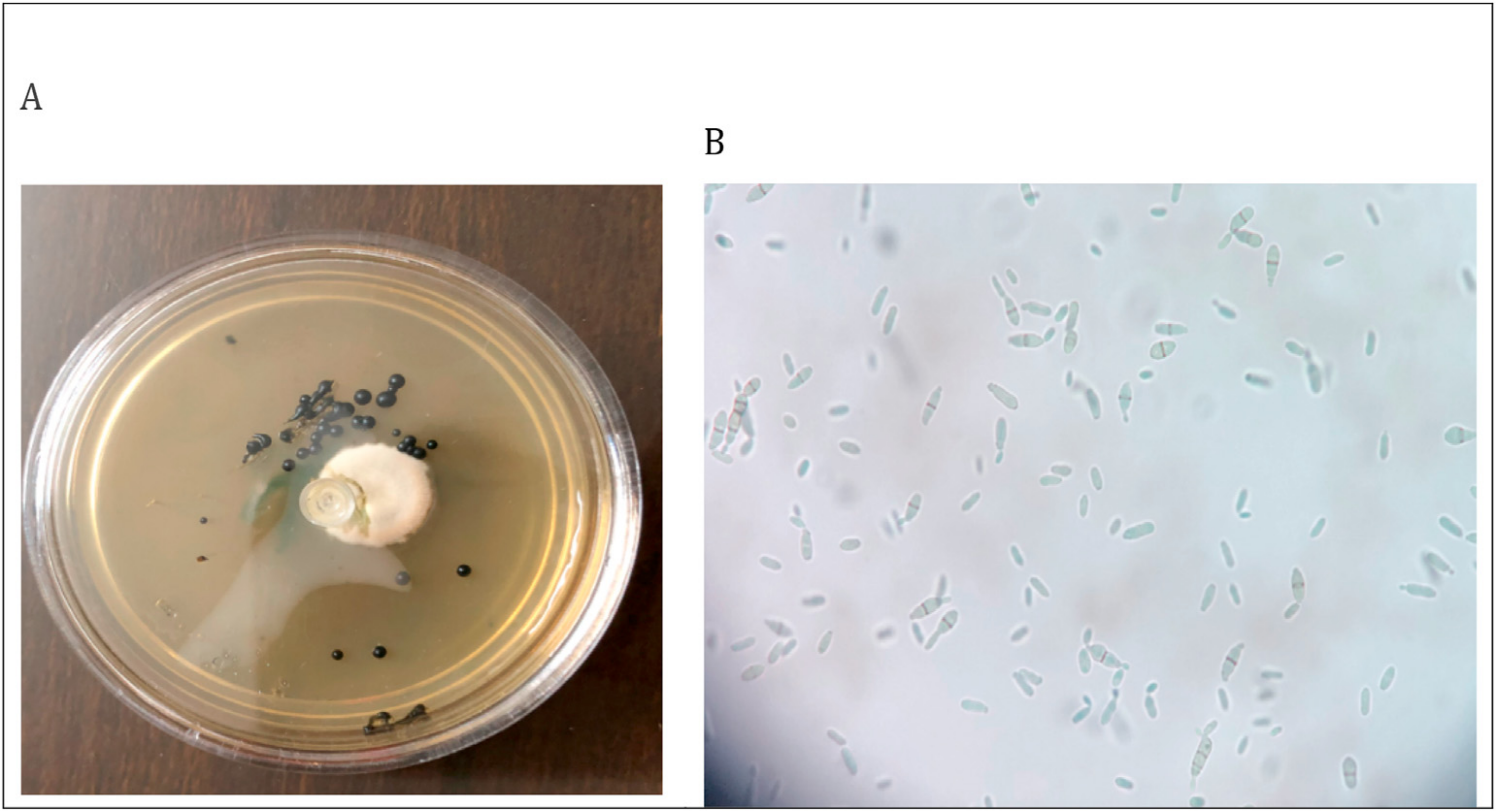
A: *Hortaea werneckii* SDA culture. B: The lactophenol cotton blue (LPCB) staining mount.

The specimen was cultured on Sabouraud's dextrose agar (SDA) with and without cycloheximide (Merck, Germany). Fungal growth was monitored on a daily basis. Identification features such as colony morphology, growth rate, and colony pigmentation were used to determine any growth obtained. Moist low growing black colonies grew on both SDA media with and without cycloheximide after 21 days of incubation at room temperature (Fig. 3, A). The lactophenol cotton blue (LPCB) staining showed two-celled brown yeasts with prominent darkly pigmented septa that taper towards the extremities forming brown to dark septate hyphae (Fig. 3, B). These features were consistent with *Hortaea werneckii*. Internal transcriber spacer region sequences (ITS) were used to confirm the strain's identity. Briefly, fungal genomic DNA was extracted from harvested colonies using the High Pure PCR Template Preparation kit (Roche, Germany) according to the manufacturer's recommendations. Then, polymerase chain reaction (PCR) was carried out bilaterally using the pan fungal primers: ITS1 (50-TCCGTAGGTGAACCTGCGG-30) and ITS4 (50-TCCTCCGCTTATTGATATGC-30) which hybridizes at the beginning of 28S rDNA (Life Technologies, Barcelona, Spain). The following thermal conditions were used: 95°C for 5 minutes, followed by 35 cycles of 30 seconds at 94 ˚C, 60°C for 45 seconds, and 72°C for 1 minute, followed by one final extension at 72°C for 5 minutes. The amplicon was sent for sequencing, which showed 100% similarity with the sequence of *Hortaea werneckii* strain R23 (accession number JQ665422.1) deposited in NCBI BLAST (http://www.blast.ncbi.nlm.nih.gov/Blast.cgi) database.

**Fig. 4:**
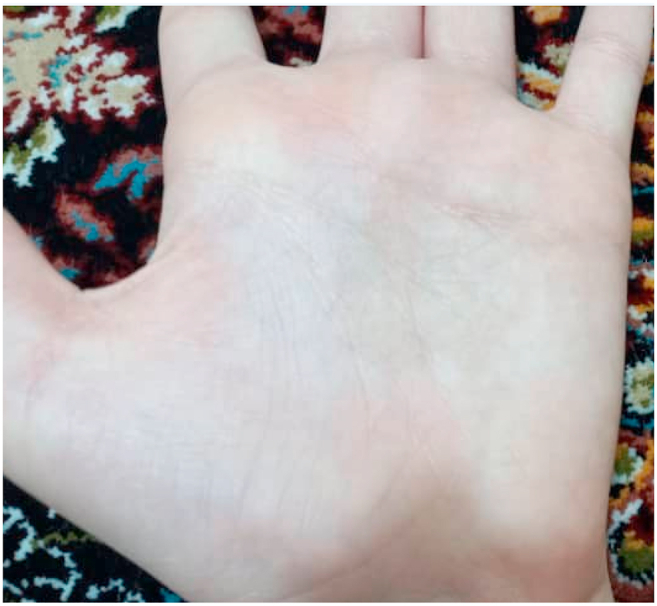
After 3 weeks treatment with 1% clotrimazole cream.

After the diagnosis of tinea nigra the patient was treated with 1% clotrimazole cream twice a day for 3 weeks with complete resolution (Fig. 4). There was no recurrence upon examination 3 months later. The patient signed a written informed consent form. All of the information was de-identified. The research ethics committee of Tehran University of Medical Sciences (TUMS) confirmed this work (the number of Ethics Committees protocol: IR.TUMS.SPH.REC.1398.197). The project was found to be consistent with Iran's ethical principles as well as national regulations and criteria for medical research.

## Discussion

Tinea nigra is an uncommon superficial fungal infection that primarily affects children and adolescents, probably due to more exposure to the fungus through contamination from an infected source (eg, soil, sand, waste, or wood) [3,4]. Our results are quite similar to those described by Bonifaz et al. in which most of the infected adolescents/children were in school-age. The majority of the patients were from rural areas in tropical and humid locations, and they resided near the sea, salt marshes, or river estuaries [1].

In a study of 12 patients conducted in Venezuela between 1972 and 2002, tinea nigra was found to be more prevalent among young people with fair skin who visited beaches [6].

Because of its ability to survive in high-salinity environments, the fungus adheres to the skin. Accumulation of a melanin-like substance present in the fungus causes hyperpigmented macules [7].

Tinea nigra seems to be less common in people with darker skin; however. this could be due to a lack of awareness about the disease [2]. Risk factors include living in or traveling to tropical areas where the organism is common [4]. Accordingly in the present report, the patient was a resident of rural communities in tropical regions (Amol, a county in the Northern Province of Mazandaran).

Due to increased global travel, patients who do not live in endemic areas should be asked if they have visited warm coastal beaches, such as tropical and subtropical beaches, mostly in Central and South America.

Pigmented patches of tinea nigra may be confused with junctional nevi, postinflammatory pigmentation, malignant melanoma, lentigines, Addison's disease, and melanosis of syphilis and pinta. Misdiagnosis of the lesions had led in the past to biopsies or surgical excisions [5]. It is not necessary to biopsy scaly lesions; a fast diagnosis can be achieved by scraping the stratum corneum with a scalpel blade and KOH preparation, which shows pigmented hyphae, and later can be confirmed by the growth of a dematiaceous mold on culture media. To the best of our knowledge, the present study is the first case of tinea nigra from Iran. This could be due to underreporting or to its actual rarity. Also, we believe that this mycosis is more common in our location, but because the clinical signs are silent, the patient is frequently unaware of the lesions and ignores them, failing to seek medical attention. Lack of awareness around this rare disease in patients, physicians and the public makes it harder to identify symptoms and receive available treatments. In addition, many people are diagnosed but not reported in other clinics. In Venezuela, 22 cases of this infection have been reported so far [6,[8], [9], [10], [11], [12]]. Alvarado et al. documented a total of 26 cases over a 30-year period in 1987 [13]. According to these authors, 57% of lesions were found on the palmar region and the rest on plantar surfaces, with an infection rate of 85% in fair skin patients. In 2008, Bonifaz et al. reviewed 22 verified tinea nigra cases during a period of eleven years (1997–2007). Twelve of the 22 patients were adults, with the others being adolescents and childrens [1].

Tinea nigra is easily treated and has a high success rate. The majority of cases respond to keratinolytic treatments such as urea, salicylic acid, and Whitfield ointment, which are used once or twice a day [1]. Miconazole, ketoconazole, bifonazole, terbinafine, and ciclopirox olamine have also been shown to have good therapy results [1]. Oral itraconazole therapy [14] has been reported, however it is not indicated for this commensal fungus. It should be mentioned that there have been reports of poor outcomes while using topical and oral griseofulvin, as well as amphotericin B cream. According to studies, prolonged therapy may be required to avoid relapse.

## Financial disclosure

This research received no specific grant from any funding agency in the public, commercial, or not-for-profit sectors.

## Transparency declaration

The authors have no conflict of interest to declare.

## References

[1] Bonifaz A., Badali H., De Hoog G.S., Cruz M., Araiza J., Cruz M.A. (2008). Tinea nigra by Hortaea werneckii, a report of 22 cases from Mexico. Stud Mycol.

[2] Charles A.J. (2009). Superficial cutaneous fungal infections in tropical countries. Dermatol Ther.

[3] Hsu L.Y., Wijaya L., Ng E.S., Gotuzzo E. (2012). Tropical fungal infections. Infect Dis Clin.

[4] Schwartz R.A. (2004). Superficial fungal infections. The Lancet.

[5] Neves J.A., Costa O.G. (1947). Tinea nigra. Arch Derm Syphilol.

[6] Perez C., Colella M.T., Olaizola C., de Capriles C.H., Magaldi S., Mata-Essayag S. (2005). Tinea nigra: report of twelve cases in Venezuela. Mycopathologia.

[7] Tilak R., Singh S., Prakash P., Singh D.P., Gulati A.K. (2009). A case report of tinea nigra from North India. Indian J Dermatol Venereol Leprol.

[8] Kerdel-Vegas F., Albornoz M.C. (1966). Tinea Nigra Report of five cases seen in Venezuela. Dermatologica.

[9] Battistini F. (1974). Dos casos de tin~a negra en el Estado Bolı´var. Com. Reunio´n Anual de la Soc Venez Dermat.

[10] Belfort E. (1973). Tinea nigra palmaris por Cladosporium sp. tercer caso observado en Venezuela. Bol Venez Dermat.

[11] Reyes O., Borelli D. (1974). Caso de tin~a por cepa peculiar de Cladosporium castellanii. Dermat Venez.

[12] Di Prisco J., Borelli D. (1973). Tinea nigra por Cladosporium species. Catellania.

[13] Alvarado J., Rodriguez H. (1987). Tin~a negra en el laboratorio de Micologı´ del Servicio de Dermatolo del Hospital Universitario de Caracas 1959–1986. Dermatologı´a Venezolana.

[14] Gupta G., Burden A.D., Shankland G.S., Fallowfield M.E., Richardson M.D. (1997 Sep). Tinea nigra secondary to Exophiala werneckii responding to itraconazole. Br J Dermatol.

